# Evaluation of a combined index of optic nerve structure and function for glaucoma diagnosis

**DOI:** 10.1186/1471-2415-11-6

**Published:** 2011-02-11

**Authors:** Michael V Boland, Harry A Quigley

**Affiliations:** 1 Glaucoma Service, Dana Center for Preventive Ophthalmology, Wilmer Eye Institute, Johns Hopkins University, Baltimore, MD, USA; 2 Health Sciences Informatics, Johns Hopkins University, Baltimore, MD, USA

## Abstract

**Background:**

The definitive diagnosis of glaucoma is currently based on congruent damage to both optic nerve structure and function. Given widespread quantitative assessment of both structure (imaging) and function (automated perimetry) in glaucoma, it should be possible to combine these quantitative data to diagnose disease. We have therefore defined and tested a new approach to glaucoma diagnosis by combining imaging and visual field data, using the anatomical organization of retinal ganglion cells.

**Methods:**

Data from 1499 eyes of glaucoma suspects and 895 eyes with glaucoma were identified at a single glaucoma center. Each underwent Heidelberg Retinal Tomograph (HRT) imaging and standard automated perimetry. A new measure combining these two tests, the structure function index (SFI), was defined in 3 steps: 1) calculate the probability that each visual field point is abnormal, 2) calculate the probability of abnormality for each of the six HRT optic disc sectors, and 3) combine those probabilities with the probability that a field point and disc sector are linked by ganglion cell anatomy. The SFI was compared to the HRT and visual field using receiver operating characteristic (ROC) analysis.

**Results:**

The SFI produced an area under the ROC curve (0.78) that was similar to that for both visual field mean deviation (0.78) and pattern standard deviation (0.80) and larger than that for a normalized measure of HRT rim area (0.66). The cases classified as glaucoma by the various tests were significantly non-overlapping. Based on the distribution of test values in the population with mild disease, the SFI may be better able to stratify this group while still clearly identifying those with severe disease.

**Conclusions:**

The SFI reflects the traditional clinical diagnosis of glaucoma by combining optic nerve structure and function. In doing so, it identifies a different subset of patients than either visual field testing or optic nerve head imaging alone. Analysis of prospective data will allow us to determine whether the combined index of structure and function can provide an improved standard for glaucoma diagnosis.

## Background

For decades, ophthalmologists have recognized a relationship between the structure of the optic nerve and its function in patients with glaucoma[1-4]. Glaucoma causes death of retinal ganglion cells and their axons, along with a deformation of the connective tissues of the optic nerve head. Thus, its structural effects can be measured both by loss of thickness of the retina in the ganglion cell and nerve fiber layers and by topographical changes of the nerve head. The alterations as measured at the nerve head combine both loss of axons and connective tissue deformation [5].

Glaucomatous functional deficits similarly result from ganglion cell loss, with defects in visual sensitivity found in the receptive fields of the damaged neurons. This relationship between optic nerve head topography and visual function is so fundamental to our understanding of the disease that it is used clinically to differentiate damage due to glaucoma from other diseases of the optic nerve head. In fact, one or both of these measures have been used as inclusion criteria and primary outcome measures in the major clinical trials of glaucoma [6-9] and in a consensus definition of glaucomatous optic neuropathy proposed for use in prevalence surveys[10].

It is logical that the two approaches, structural and functional, should give information that is highly correlated, since both depend on atrophy of retinal ganglion cells whose anatomical and physiological features are known. On the other hand, data from clinical trials have failed to confirm a strong concordance between changes in optic nerve and visual field criteria. In the Ocular Hypertension Treatment Study, for example, treated subjects met both field and optic nerve criteria for change only 8% of the time, while 42% reached the visual field end point alone and 50% reached the optic disc end point alone[11]. The lack of perfect correlation between structural and functional diagnostic criteria may have one or more explanations. For example, it is known that the variability in testing differs between the two methods, which could by itself produce poor clinical correlation. Alternatively, the two approaches might contain different types of information about the presence or absence of glaucoma, which, while correlated by the anatomy of the visual system, nonetheless are expressed in different ways with different timing from individual to individual. Thus, using either of these two measures alone as a "gold standard" for glaucoma diagnosis may be a flawed approach.

Hence, the merging of the two sets of data could provide important corroboration that true abnormality had developed or was progressing. This has not been previously done in optimal fashion for a variety of reasons. First, there has been no attempt to combine quantitatively structural and functional deficits using continuous probabilities of abnormality for each measure. Rather, specific cut-off criteria at extreme probability levels are given in current imaging or field analysis. These values have been chosen to maximize the specificity of each test, though at the cost of sensitivity. Second, there has been no consensus regarding a map between the position of a field deficit and corresponding structural changes to the NFL thickness or optic nerve head topography. Some proposed models for matching structure and function used probabilistic approaches that produced findings inconsistent with known anatomic facts--linking superior visual field defects to superior optic disc abnormality, for instance[12-16]. Third, it is possible that there is temporal dissociation in structural and functional abnormality development. While NFL photographs showed abnormality in many cases earlier than manual visual fields[17], it has recently been proposed that much of the apparently earlier change in structure derives from greater variability in field test data[18]. Regardless of the actual nature of temporal discontinuity in changes to structure and function, use of both together better reflects the fact that glaucomatous optic neuropathy displays changes in both structure and function.

We believe a single measure combining these elements is an improvement for 4 reasons. First, quantitative measures of optic nerve structure and function are increasingly available and improving in quality. Second, the discordance between simultaneous progression suggests that both structure and function contain complementary information. Third, a reliable mapping of visual field points to the optic nerve head and nerve fiber layer is available. Finally, modulation of the variability of structural and functional tests by emphasizing those that are anatomically related will reduce the overall variability of the system. To test the hypothesis that a combined measure of optic nerve structure and function might be able to detect glaucoma, we designed the Structure Function Index (SFI) as a model unifying retinal ganglion cell structure and function. The link between the two was created using our knowledge of retinal nerve fiber layer anatomy. We then analyzed the characteristics of the model and compared it to tests of structure and function alone in terms of distinguishing eyes with glaucoma from eyes of glaucoma suspects.

## Methods

This work was approved by the institutional review board of the Johns Hopkins University School of Medicine and adhered to the tenets of the Declaration of Helsinki.

### Study Populations

Evaluation of the SFI required three groups of subjects: 1) A *reference *group of glaucoma suspects to define "normal" values of tests, 2) a separate but identically defined group of glaucoma *suspects *and 3) a group with *glaucoma*. The latter two groups were used to compare the SFI to current diagnostic methods.

We identified subjects for the *reference *and *suspect *groups using billing data to select patients seen by the Glaucoma Service of the Wilmer Eye Institute between 1999 and 2007 with a diagnosis of glaucoma suspect (ICD-9 code 365.0x). The criteria for diagnosis of glaucoma suspect and glaucoma were based on the clinical judgment of glaucoma specialists using medical history, past records, and a comprehensive clinical evaluation including visual field testing and optic nerve examination. We further refined this list to include those subjects who, on the same day, had both visual field testing (SITA-Standard 24-2) classified as 'Reliable' by the Humphrey Field Analyzer (HFA, Carl Zeiss Meditec, Dublin, CA) and optic nerve imaging using the Heidelberg Retina Tomograph (HRT, Heidelberg Engineering, Vista, CA) with image quality that was at least 'Acceptable' according to the HRT software. These tests were chosen because they were most commonly used by the Wilmer Glaucoma Service during this time period. Subjects were also excluded if they ever had a diagnosis of glaucoma (ICD-9 codes 365.[1-9]x) in our database. Finally, the group identified as glaucoma suspects was required to achieve a stage of 0 or 1 on the Glaucoma Staging System[19]. This search returned 1558 eyes. Data from right and left eyes were analyzed separately so a given patient was allowed to contribute both eyes to the *reference *group. Rather than treating tests from right and left eyes merely as mirror images, we kept them separate in order to account for any differences based on laterality, due to testing order for example. One thousand of these subjects were randomly selected as our *reference *population and the remaining 558 were placed in the *suspect *population to be used in evaluating the SFI.

Similarly, glaucoma cases were identified as those subjects with a diagnosis of open angle glaucoma (ICD-9 365.11), a 'Reliable' visual field and at least 'Acceptable' quality HRT on the same day.

In cases where both eyes were available for a particular subject in the *suspect *or *glaucoma *groups, the eye with the worse HFA mean deviation was used in the analysis described below. This was done since clinicians would likely classify a subject as glaucoma if one eye were affected, and we did not wish to include fellow eyes with lesser or no damage in the glaucoma group. After this selection process, we had 499 eyes from suspects and 895 eyes with glaucoma.

To validate the diagnoses obtained using billing data, we randomly selected the clinical chart records of 100 subjects identified as glaucoma suspects and 103 subjects identified as having open angle glaucoma. Of the 100 records reviewed for glaucoma suspects, 97 supported the billing diagnosis based on the clinician's written assessment of the patient. Of the remaining three, one had angle closure glaucoma, one had no glaucoma diagnosis in the chart, and one had neurological disease. Of 103 records reviewed from those subjects with billing codes for open angle glaucoma, 99 had glaucoma (one angle closure), 3 were suspects and 1 had non-glaucomatous optic nerve disease. Based on this review, we can therefore estimate that only about 4% of billing codes do not accurately reflect the clinician's assessment. Furthermore, the number of misclassified individuals is similar in each group (suspect and glaucoma) so there should be no net bias of the classifier performance.

### Defining the Structure Function Index

In brief, the SFI is calculated in three steps: 1) calculate a probability of abnormality for each point in the visual field, 2) calculate a probability of abnormality for each sector of the optic disc, and 3) combine those probabilities with the probability that a visual field point and optic disc sector are linked by retinal nerve fiber layer anatomy. As defined, the SFI produces one probability of structure-function abnormality for each point tested in the visual field. To allow the SFI to be compared to current diagnostic criteria, we analyzed these individual pointwise SFI values in a manner similar to the Glaucoma Hemifield Test[20].

### Definition of Reference Values

To calculate the probability of abnormality for each visual field point, we require reference data for each of those points. The probabilities of abnormality reported by the standard output of the HFA include only a small number of discrete cutoffs near the far end of the probability distribution function (p = 5%, 2%, 1%, 0.5%)[21]. They therefore contain no information about probabilities of abnormality less than 95% and are based on reliable tests of persons with a normal eye examination. To generate a continuous probability of abnormality at each point, we created empiric distributions for the HFA Total Deviation at each point in the 24-2 visual field for right and left eyes separately. These distributions were created using pointwise values from 500 right and 500 left eyes in the reference group defined above. Total Deviation values were used because they include correction for age-related decline in visual sensitivity. We chose not to use Pattern Deviation values as they include a correction for diffuse loss that we believe will be unnecessary with the SFI because diffuse field loss not associated with corresponding nerve damage will be discounted to a large degree in the SFI calculation.

**Figure 1:**
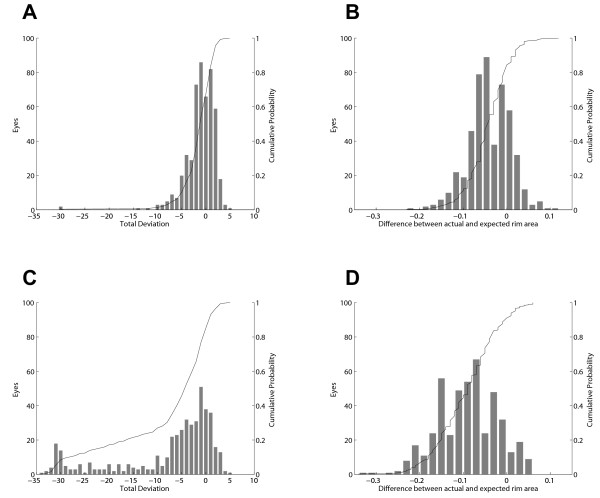
**Example distributions of structural and functional data**. The distribution of HFA Total Deviation at the point 3 degrees to the right and 15 degrees above fixation (A), and the distribution of the difference between measured and predicted rim area for the inferior-temporal sector of right eyes in the reference group (B). The same values from the glaucoma group are plotted in C and D. The line in each figure represents the cumulative probability function (CPF) for each distribution and helps to demonstrate the probability of normality concept used in the SFI calculation. Values to the left have low probabilities of normality (low values of the CPF) and values to the right have high probabilities of normality (high values of CPF).

Second, we extracted HRT data for the 1000 reference eyes from our clinical database and generated empiric distributions for the difference between measured rim area and the rim area predicted by Moorfields regression analysis (MRA)[22] in each of the six sectors reported by the HRT. The Moorfields predicted rim area was used in this way as it includes normalization for both disc area and age. All HRT data were analyzed using the HRT-3 software which has an expanded database of normal subjects[23]. Example distributions of HFA total deviation and HRT rim area difference values for our reference population are shown in Figure 1A and B. Also shown in Figure 1 is the cumulative probability function (CPF) for both measures. We use the CPF to define the probability of normality of a particular value. Less negative values of total deviation or difference from expected rim area would be expected in normal subjects and so they have higher values on the CPF (higher probability of normality). In this way, we obtain a continuous function describing the degree of abnormality of each measurement. Corresponding distributions for the 895 subjects in the glaucoma group are shown in Figure 1C and 1D.

### Linking Points in the Visual Field to Points on the Optic Disc

**Figure 2:**
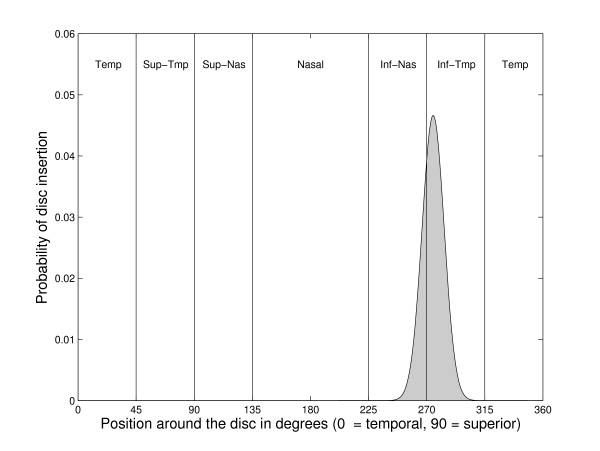
**Probability of association between a visual field point and the optic disc**. Probability of insertion around the optic nerve head for a visual field point 3 degrees to the right and 15 degrees above fixation in a right eye. The vertical lines indicate boundaries between the six standard sectors analyzed by the HRT. Calculating the area under the curve for each sector, the probability of this point being associated with the inferior-nasal sector is 26% and with the inferior-temporal sector is 74%.

The third step in calculating the SFI links rim area to each field point based on a map developed using nerve fiber layer defects seen in red-free photographs[24]. This approach bases the relationship of structure and function on known anatomy rather than on statistical correlation alone. Briefly, the Garway-Heath group started with fundus photographs that depicted focal nerve fiber layer defects. They then used an overlay of visual field test locations to determine which field points would have been affected by each defect. Finally, they measured the angle of insertion of the defect at the nerve head. After reviewing 69 photographs in this way, the output of this process was a list of disc insertion angles for each point in the visual field. In the published version of this map, the linkage of field position and rim sector are depicted as absolute, but we extended this approach by estimating the uncertainty regarding where each field point maps to the optic disc. To do this, we obtained the mean and variance for disc insertion angle for each field point, as measured by Garway-Heath et al. (personal communication). These data were then used to estimate the probability that a particular visual field point is anatomically linked to a particular optic disc sector, by assuming normal distributions for each field point and summing the area under this empirically derived probability distribution in each disc sector. The process of linking a visual field point to HRT sectors is shown graphically in Figure 2.

### Quantitative Combination of Structure and Function

Once the reference values are available for the tests of structure, function, and for the anatomical relationship between the two, the SFI is calculated for each of the 52 points outside the blind spot in the 24-2 pattern visual field by multiplying the three probabilities described above together and summing over all HRT sectors:

(1) $$SFI=\sum_{\text{All Sectors}}P(\text{field})\cdot P(\text{sector})\cdot P(\text{anatomy})$$

Where P(field) is the probability that the visual field point is abnormal (one minus the cumulative probability value defined above), P(sector) is the probability that an HRT sector is abnormal (using the cumulative probability function defined above), and P(anatomy) is the probability that the visual field point is linked to that sector of the nerve. Using this formula, the SFI will be close to 0 in cases where the probabilities of abnormality for the field and disc are small or when the two are unlikely to be linked by RNFL anatomy. It is also the case that when there is a modest probability of a defect in function and a modest probability of a defect in structure, the fact that the two are in linked by RNFL anatomy increases the value of the SFI compared to when they are not linked.

**Figure 3:**
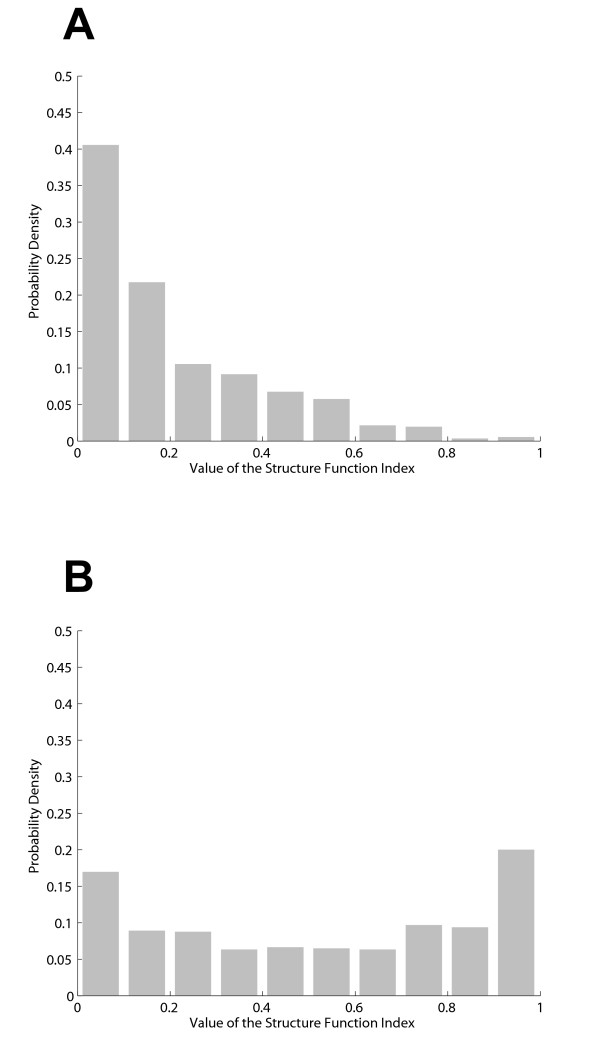
**Distribution of Structure Function Index values**. Distribution of values for the structure function index at the point 3 degrees to the right and 15 degrees above fixation in right eyes of the reference (A) and glaucoma (B) groups.

As an explicit example of how the SFI is calculated at each corresponding visual field point, assume we start with a visual field total deviation of -7 at the point 3°,15°. The probability that this value is "normal" can be obtained using the cumulative distribution shown in Figure 1A. A total deviation of -7 falls at 8% on this curve indicating that a value this low would only be expected in 8% of the reference population (i.e., there is a 92% chance it is abnormal). Next, assume that the inferior-temporal HRT sector from the same patient has an actual rim area of 0.25 mm^2 ^and that the area predicted by Moorfields regression is 0.20 mm^2^. The difference between these two measures (0.05) falls at the 98% point on the distribution shown in Figure 1B (a 2% chance it is abnormal). Probabilities are similarly calculated for each HRT sector. To complete our example, assume that the probability for the inferior-nasal rim area difference is 84% (16% chance it is abnormal). We then need to know how likely it is that the visual field point is anatomically linked to each of the HRT sectors. For this, we use distributions like the one in Figure 2 which shows that visual field point (3°,15°) is associated with the inferior-temporal nerve sector in approximately 74% of eyes and with the inferior-nasal sector in 26%. Since the probability that this visual field point is linked to the other four HRT sectors is near zero, those sectors make no contribution to the SFI calculation (i.e., they add only zero terms to the sum in Equation 1). The probability values found above are then used to calculate the value for the SFI at this point: (1-0.08)(1-0.98)(0.74) + (1-0.08)(1-0.84)(0.26) = 5.4%. This number then represents the probability of an abnormal structure-function relationship at this point in the visual field. The distribution of SFI values in the suspect and glaucoma groups at a single point are shown in Figure 3. SFI values are calculated for all locations in the same way.

### The SFI Hemifield Test

**Figure 4:**
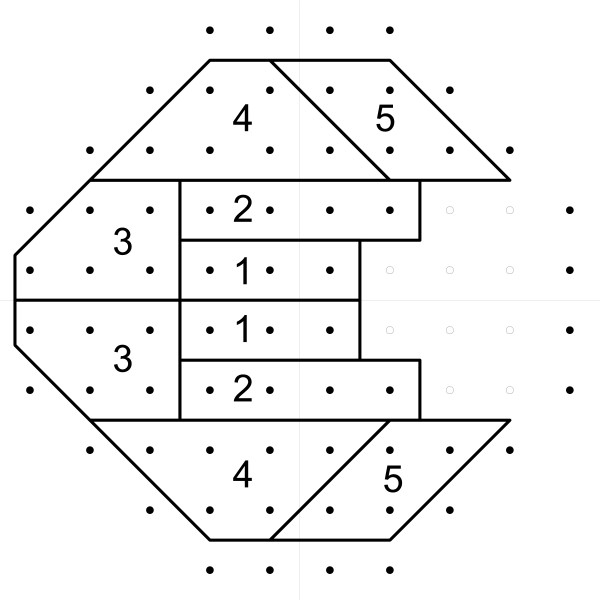
**Regions for the Structure Function Index Hemifield Test (SFI-HT)**. Regions defined for the Structure Function Index Hemifield Test (SFI-HT) in a visual field from a right eye.

As defined above, the SFI produces values for an overall probability of abnormality corresponding to each point in the visual field. Since it is known that glaucoma often affects one hemifield (upper or lower) differentially, due to the presence of the horizontal raphe, we implemented an SFI Hemifield Test (SFI-HT) which is an algorithm similar to the Glaucoma Hemifield Test (GHT) used in the HFA[20]. Using the same 3 to 6 point clusters, matched above and below the horizontal meridian, as defined in the GHT (Figure 4), we calculated a score for each of the 10 clusters (5 superior and 5 inferior) using:

(2) $$\text{Region Score}=\sum_{\text{All points}}\frac{1}{10(1-\text{SFI})}$$

As the SFI values within a region become closer to 1 (100% probability of abnormality), the value of the region score increases. The formula above is derived directly from that used for calculating region scores in the GHT. Because this value can reach very high levels as the SFI value becomes very small, we limited the score for any one point to a maximum of 100 (SFI value of 99.9%). The maximum score for a given region depends on the number of points included (3 to 6) and can therefore range from 300 for region 1 to 600 for region 4. The difference in the score between paired superior and inferior regions was then used to identify defects that might be due to glaucoma.

To define a reference distribution for the difference in hemifield score for each cluster pair, we again used the 1000 subjects in the reference population defined above. We calculated the SFI at each point in the visual field, calculated the region scores, and then calculated the differences between corresponding superior and inferior region pairs. Recognizing that upper to lower differences disappear when damage is severe in both hemifields, we also calculated the sum of all SFI-HT region scores. This value was included in the analysis to account for eyes with damage too diffuse to produce a significant superior-inferior difference in scores. Based on the 500 right and 500 left eyes, we were able to generate empiric distributions for the five differences in hemifield region scores and for the sum of region scores. When subsequently analyzing the glaucoma suspect and glaucoma subjects, we determined which of these six values was most statistically abnormal by comparing each to the corresponding empiric distribution from the reference population. To allow for receiver operating characteristic (ROC) analysis to be performed, each subject was summarized with the single largest probability of abnormality among its five regional score differences (Figure 4) and sum of all 10 individual region scores (5 upper and 5 lower in Figure 4). The output of the SFI-HT is therefore a value between 0 (no chance of being abnormal) and 1 (100% chance of being abnormal).

### Statistical Analysis

The demographics of the glaucoma and glaucoma suspect groups were compared using the t-test for age, Fisher's exact test for sex, and the chi-square test for racial background. The data for race are based on self-report as recorded in the Johns Hopkins Hospital patient registration system.

The SFI and the SFI-HT were calculated for each eye in the glaucoma group and for those eyes in the glaucoma suspect group that were not used as the reference population. Receiver operating characteristic analysis was then performed to characterize the ability of the SFI to distinguish eyes with glaucoma from eyes of glaucoma suspects. The probability of the most abnormal difference in SFI-HT region scores or of the sum of all SFI values was used as the summary statistic for the SFI with the clinical diagnosis serving as the "true" classification. To compare the SFI-HT to existing diagnostic criteria that rely on structure or function alone, we performed ROC analysis using the HFA mean deviation (MD) and the difference between actual and predicted rim area from the HRT. We also calculated the sensitivity and specificity of the HFA GHT and the categorical HRT Moorfields classification (MFC). The results of these two tests were considered positive for glaucoma when they were reported as "Outside Normal Limits". Areas under the ROC curves were compared using the method of Hanley and McNeil[25] and confidence intervals for sensitivity and specificity were calculated using the method described by Ross[26]. The "optimal" operating point of the SFI ROC curve was chosen as that value of the SFI-HT that produced the point closest to 100% sensitivity and 100% specificity.

Visual field and HRT data were analyzed using Matlab (version 7.11.0, The Mathworks, Inc., Natick, MA) and statistical analyses were performed using Matlab, and R version 2.11.1[27] with packages Hmisc[28] and ROCR[29].

## Results

**Figure 5:**
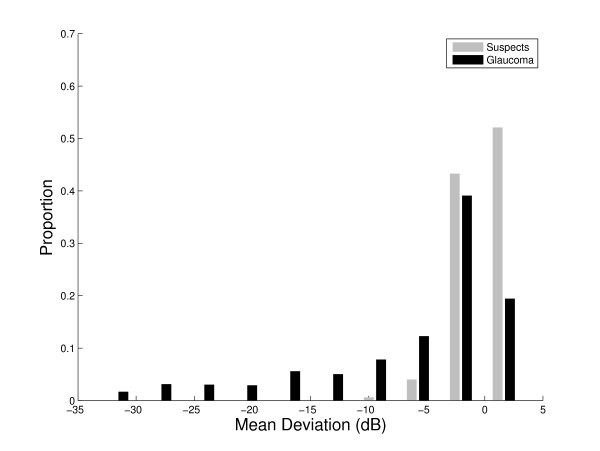
**Distribution of visual field mean deviation**. Frequency distribution of visual field mean deviation (MD) in 499 glaucoma suspects and 895 glaucoma patients.

The test group of glaucoma suspects was significantly younger and contained a higher proportion of females than the glaucoma patients (Table 1). The fact that glaucoma suspects are younger than those with the disease is expected from the increasing incidence of glaucoma with age. The higher proportion of women among the suspects is not expected, as the prevalence of open angle glaucoma is similar between sexes. The finding may derive from the known tendency for women to make more visits to physicians than men in the United States and from the fact after age 65 women outnumber men by 60% to 40%[30]. These differences in the two groups are not expected to affect subsequent analysis, since we used measures of structure (difference from HRT Moorfields predicted rim area) and function (HFA total deviation) that account for the age of the patient. The Moorfields regression function used by the HRT to predict rim area also accounts for disc area, which may be slightly larger in men[31]. There are no reported differences between men and women on visual field testing, so we do not expect the higher proportion of women in the suspect group to bias classification. Also, as expected, the mean deviation (MD) and pattern standard deviation (PSD) were significantly larger in magnitude in the glaucoma group (Table 1). Furthermore, 95% of the suspect group had a MD value greater than -4 dB. In the glaucoma group, 98% had an MD greater than -25 dB (Figure 5).

**Table 1:** Characteristics of the glaucoma suspect and glaucoma patient groups.

	Glaucoma Suspects (n = 499)	Glaucoma Patients (n = 895)	p-value
Age (years)	52(15)	65(13)	<0.001
Sex (% female)	65	52	<0.001
Race (%)			0.18
White	69	70	
Black	17	17	
Asian	3.4	3.2	
Hispanic	1.8	0.5	
Indian	0.8	0.1	
Other	4.0	5.9	
Unknown	3.5	3.3	
HFA MD (dB)	-0.46(1.7)	-6.3(8.3)	<0.001
HFA PSD (dB)	1.8(1.0)	4.9(4.0)	<0.001

As part of the review of clinical records for 100 of the glaucoma suspects, we determined that 48% were suspects based on the appearance of their optic nerve, 23% based on a history of elevated intraocular pressure, 11% based on family history, 6% based on the appearance of their anterior chamber angle, 4% based on their visual field, and 8% for other reasons. A cup-disc ratio was recorded for 189 eyes from these 100 suspects and the mean of these values was 0.49 with a standard deviation of 0.17. For comparison, a documented cup-disc ratio was found for 184 eyes of the 103 subjects reviewed in the glaucoma group and the mean was 0.76 with a standard deviation of 0.20.

**Figure 6:**
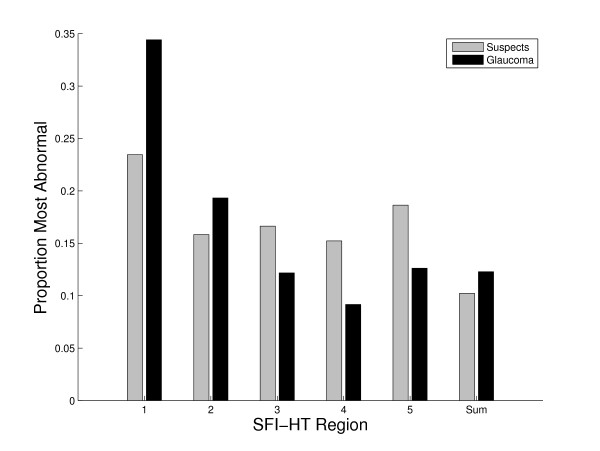
**Distribution of the most abnormal SFI-HT regions**. Distribution of the statistically most abnormal difference between corresponding superior and inferior SFI-HT regions for 499 suspect and 895 glaucoma eyes. The SFI-HT region numbers correspond to the regions in Figure 3 and Sum refers to the sum of all region scores (to account for diffuse loss).

SFI values were calculated for each member of both the suspect and glaucoma groups. The 52 SFI values for each subject were then summarized with a single value using the SFI-HT. As described above, the SFI-HT is a single probability value representing the most statistically abnormal difference between the upper and lower members of the 5 region pairs or the overall sum of region scores. To determine which hemifield regions are most likely to be abnormal in each group, the frequency with which each of these six values (five hemifield differences and sum of all regions) was most statistically abnormal is shown in Figure 6. Regions 1 and 2 and the sum of all SFI values were all somewhat more likely to be the most abnormal in subjects with glaucoma than they were in glaucoma suspects. The fact that these anatomic regions are more likely to be abnormal in persons with glaucoma may therefore have added significance for glaucoma diagnosis.

**Figure 7:**
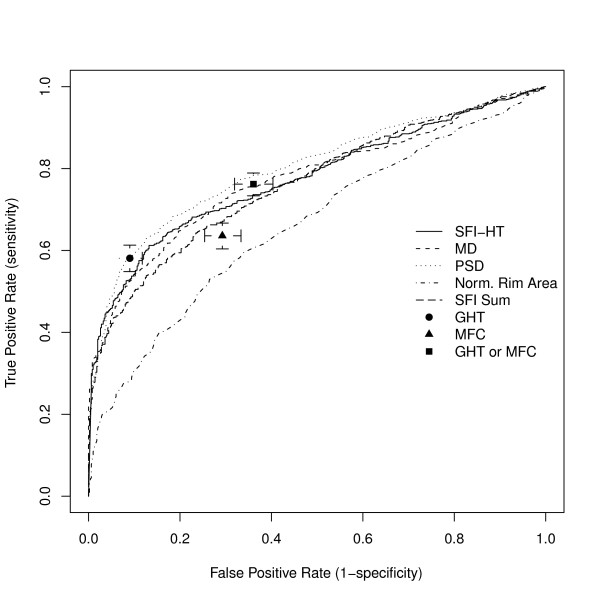
**Receiver Operating Characteristic analysis**. Receiver Operating Characteristic (ROC) analysis of the Structure Function Index Hemifield Test (SFI-HT), Humphrey Field Analyzer Mean Deviation (MD), pattern standard deviation (PSD) and difference between predicted and actual Heidelberg Retina Tomograph rim area. Each test was used to distinguish a group of 499 eyes from glaucoma suspects from a group of 895 eyes with glaucoma. The areas under the SFI-HT, MD, PSD, and HRT MRA curves are 0.78, 0.78, 0.80, and 0.66 respectively. The performance of the HFA Glaucoma Hemifield Test (GHT) and HRT Moorfields Classification (MFC) are also shown along with the performance of defining as abnormal any subject with either of these tests outside normal limits. Error bars represent 95% confidence intervals for sensitivity and specificity.

We assessed the ability of the SFI-HT to distinguish eyes with glaucoma from those that were suspicious for disease using ROC analysis (Figure 7). The area under the ROC curve for the SFI-HT was 0.78, indicating fair performance as a classifier. The area under the ROC curve for HFA MD was 0.78, for PSD 0.80, and the area under the curve for the normalized rim area was 0.66. Only the area under the curve for the normalized rim area was statistically different than that for the SFI (p < 0.001). As categorical variables, the GHT and MFC produced single values for sensitivity and specificity - 58% and 91% respectively for the GHT and 64% and 71% for the MFC. As expected, declaring a subject abnormal if either the GHT or MFC was abnormal resulted in higher sensitivity than either test alone (76%) but at the cost of lower specificity (64%). We also tested the mean value of the SFI for each subject using ROC analysis and found that it produced an area under the ROC curve (0.76) that was not significantly different from the SFI-HT (0.78), suggesting that the extra computation of the SFI-HT may not be necessary (data not shown).

**Figure 8:**
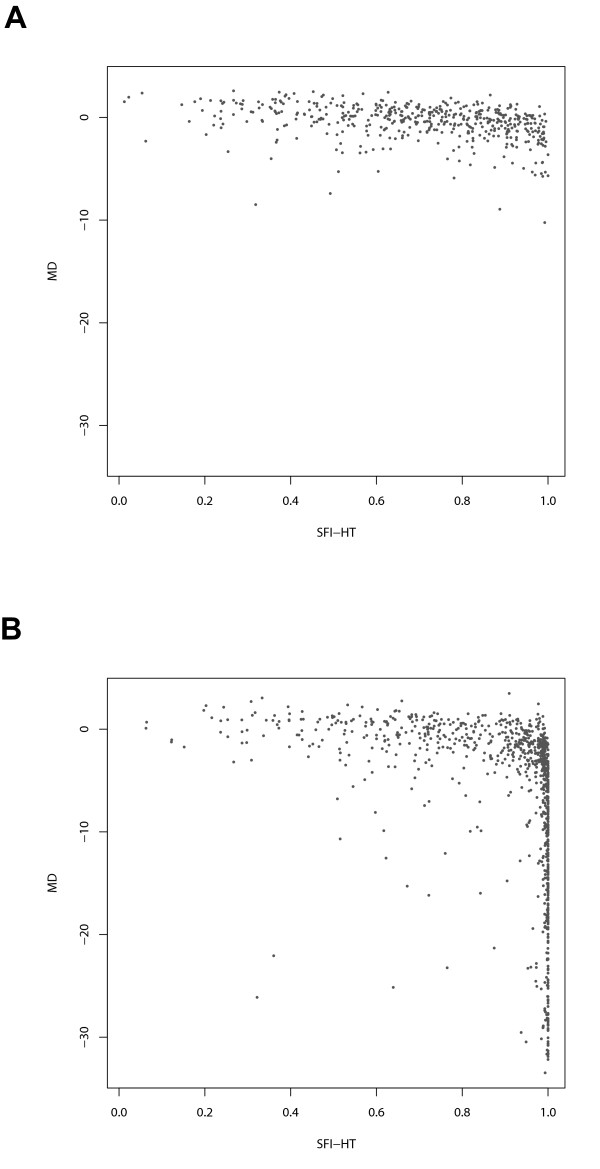
**Relationship between the SFI-HT and visual field mean deviation**. Relationship between the Structure Function Index Hemifield Test (SFI-HT) and visual field mean deviation (MD) in the glaucoma suspect group (A) and the glaucoma group (B).

**Figure 9:**
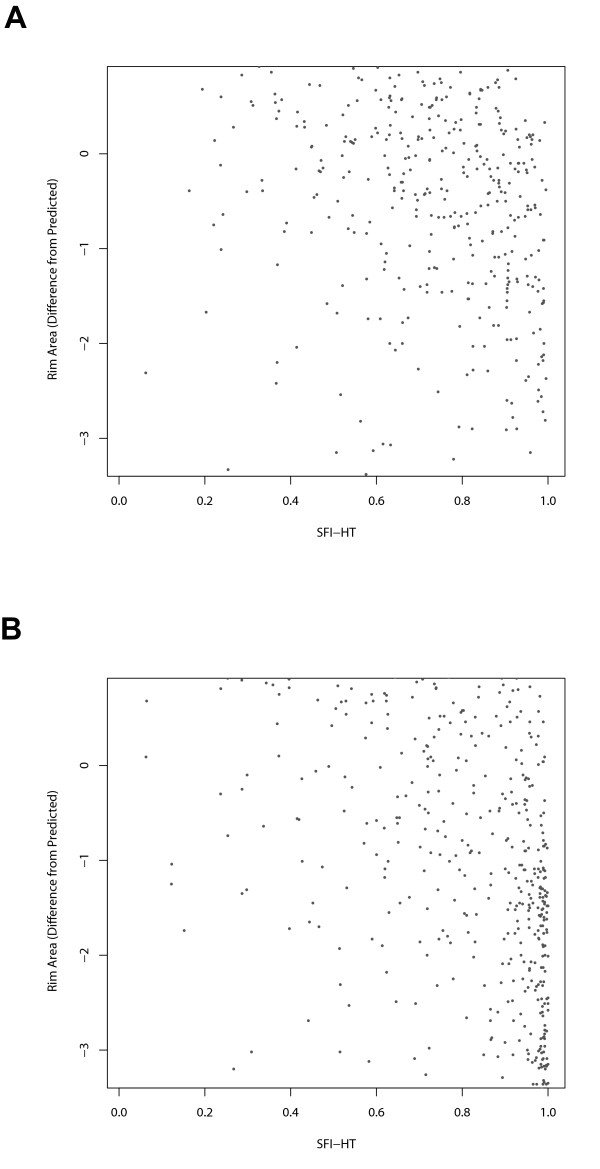
**Relationship between the SFI-HT and values for difference from predicted HRT rim area**. Relationship between the Structure Function Index Hemifield Test (SFI-HT) and HFA difference from predicted rim area in the glaucoma suspect group (A) and the glaucoma group (B).

**Figure 10:**
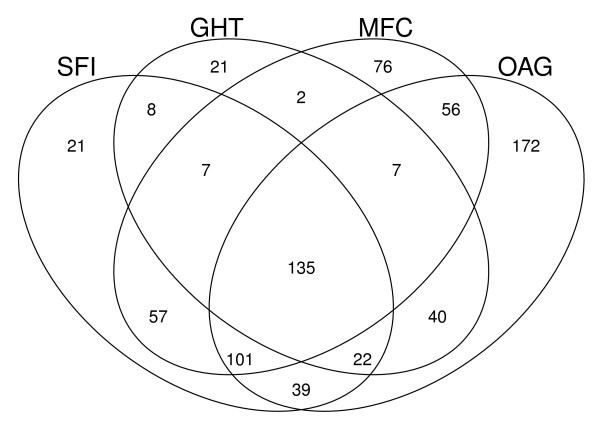
**Diagnosis of glaucoma by different methods**. Venn diagram of positive diagnostic tests in the 1058 members of the suspect and glaucoma groups with visual field mean deviation greater than -5dB. SFI = Structure Function Index Hemifield Test, GHT = Glaucoma Hemifield Test, MFC = Moorfields classification, OAG = clinical diagosis.

To further characterize the performance of the SFI and other diagnostic tests, we visualized the values of the tests pairwise for both the suspect and glaucoma groups. Examples include plots of visual field MD (Figure 8) and HRT difference from predicted rim area (Figure 9) versus corresponding values of SFI-HT. While there is a less pronounced relationship between rim area and the SFI (Figure 9), note that at low values of MD (Figure 8), the SFI-HT is distributed across a wide range of values and that almost all of the subjects with high values of MD also have high values on the SFI-HT. Because the group with mild disease represents the greatest diagnostic challenge, we created a Venn diagram showing the number of subjects with "mild" disease (MD > -5dB) declared positive by each test (Figure 10). This subset of the suspect and glaucoma groups included 1058 (out of 1394) eyes and we considered the clinical diagnosis as a fourth test of glaucoma. Of the 572 subjects diagnosed with glaucoma by a clinician, 135 (24%) were positive by all three other tests (GHT, MFC, SFI). In a similar diagram including only those subjects with severe disease (MD < -10dB), the fraction positive by all tests was 84% (176 out of 210). Other interesting aspects of the mild group include the fact that 172 subjects were positive by clinical diagnosis only and 118 were positive by only one of the other three tests (21 SFI, 21 GHT, 76 MFC).

## Discussion

These results characterize a potentially new approach to the diagnosis of glaucoma. Whereas the clinical diagnosis of glaucoma requires that defects in optic nerve structure and function should be found together, there is currently no test available to quantitatively combine measures of both structure and function. We propose the SFI as such a test. We have explicitly defined it to overcome some of the limitations of current testing. First of all, it utilizes continuous probabilities of abnormality rather than relying on the highly specific "abnormal" determinations of each device. The use of continuous probabilities has also been proposed for analysis of visual field data[32]. Relying on this feature alone might make the SFI prone to false positive results, however. To overcome this, the SFI explicitly modulates defects in structure and function by requiring an anatomic relationship between the two, thereby augmenting defects that would not reach statistical significance on either test alone.

The results presented above show some desirable characteristics of the SFI. We see that the values of the SFI in the reference population are heavily skewed toward the expected (lower) values (Figure 3A). At the same time, the values for the glaucoma population are more spread out between low and high values (Figure 3B). Since Figure 3 depicts data for a single visual field location, one would expect such a distribution in the glaucoma group, as a particular location may not be affected in some individuals. A similar comment can be made about Figure 8. The fact that the SFI values are widely distributed for subjects with "mild" disease (based on visual field) is expected as this group contains both normal subjects and those with varying degrees of early damage. We are now investigating through the use of longitudinal data whether those with higher levels of SFI will show more rapid progression, thereby validating the index. This finding would not be as significant if the subjects with more severe field loss were also widely distributed in terms of the SFI. The fact that they are not suggests that the SFI is not simply a random number generator.

The ROC data also suggest that the SFI is a useful synthesis of structure and function. First of all, while the total area under the curve is not significantly different from those of MD and PSD, it does show higher sensitivity at the highest specificity values. The Venn diagram in Figure 10 showing the classification of subjects by each test also helps with the interpretation of the ROC curves. While all tests are highly correlated in severe disease (data not shown), there is significant disagreement for those with the mildest disease shown in the figure. In other words, each test is classifying (or misclassifying) different subjects on the right side of the ROC plot, the zone containing those with mild disease. As always, the lack of an accurate test for glaucoma makes it difficult to compare new to existing methods. A determination of which diagnostic test (including the clinician) is correct is not possible using data from a single point in time. The ultimate evaluation of the SFI (or any glaucoma test) will be longitudinal studies, now ongoing, in which development of initial injury will be compared among all diagnostic tests.

The areas under our ROC curves are lower than some prior studies using other patient populations and other diagnostic tests. This is likely due to the fact that we purposefully studied a challenging classification problem by attempting to distinguish a group of glaucoma suspects from those classified as glaucoma. Studies that evaluate the ability to discriminate between eyes with glaucoma and normals would be expected to find more striking differences, though this comparison does not duplicate the problem encountered by clinicians. To that end, we chose to use glaucoma suspects to define our normal values since they are, in fact, the group that clinicians are most often forced to differentiate from patients with early glaucoma. It is seldom a dilemma in clinical practice to distinguish a patient with low glaucoma risk (no family history of glaucoma, normal optic nerve appearance, normal visual field, normal IOP) from someone with manifest disc or field change. On the other hand, it is a frequently encountered problem to determine which patients with clear risk factors (strong family history, "suspicious" discs, non-specific field changes, elevated IOP) have disease and which do not. By choosing to use suspects as our reference group, we are therefore making the classification problem more difficult, but also more applicable to clinical practice.

Previous research on combining measures of structure and function used regression models that included variables from various optic nerve analyses and from automated perimetry[33]. Subsequently, investigators applied machine learning techniques to combined structural and functional data[34-37]. While some of these studies reported an improvement of one kind or another in the detection of glaucoma, none included the steps of explicitly combining the structural and functional data using knowledge of nerve fiber layer topography or the superior-inferior difference in glaucoma damage. It may also be possible for a machine learning classifier to "learn" nerve fiber layer anatomy given enough training data. However, training a system in this way will always be hampered by the fact that empiric data contain correlations between structural and functional defects that are not due to a cause and effect relationship. For example, when significant damage has occurred, correlations between disc rim loss and decreased field test sensitivity will occur simply because all points are functionally depressed and not necessarily because the two are linked by ganglion cell anatomy. In other words, when the disc and field are both severely, rather than focally damaged, one will be able to find correlations between disconnected areas like superior field points and superior optic nerve parameters. Furthermore, most machine learning classifiers represent "black boxes" that model knowledge in ways that are not easily understandable. By explicitly including our knowledge of the anatomic basis for structure-function correlations in glaucoma, we avoid the need to "teach" classifiers how structure and function are related by anatomy in glaucoma. On the other hand, machine-learning approaches may provide the benefit of discovering alternative relationships between structure and function in glaucoma, though this benefit remains to be seen.

Another area of investigation that has some relationship to what we present here is the modeling of structural and functional changes in glaucoma. Starting with the assumption that changes in sensitivity at a particular point in the visual field should correlate closely with changes in nerve fiber layer thickness or the number of ganglion cells, both Harwerth *et al*[38]. and Hood *et al*[39]. have proposed linear models of this relationship. Both have shown significant correlation between structural and functional measures in both animals and humans and support the concept that glaucoma produces changes in both. While these models are useful for understanding local relationships between loss of ganglion cells and loss of visual sensitivity, they have not yet been shown to have application to diagnosis of disease. Furthermore, the variability in the data used to create the models and the subsequent uncertainty in the models themselves suggests that it will be difficult to apply them to individual patients. By emphasizing anatomically meaningful relationships, the SFI may therefore be a useful tool to bridge the gap between work on local correlations between structure and function and the significant variability that exists within each test alone.

Analysis of our study is clearly limited by the fact that it was carried out retrospectively. Specifically, the diagnostic criteria used by the clinicians as expressed in the billing code data were not standardized, so there is potential variability in diagnostic classification. We confirmed the validity of our diagnostic coding by reviewing a subset of charts, revealing a small error rate compared to the documented clinical impression and no evidence for bias in misdiagnosis favoring either group. Any study of glaucoma faces the difficulty that diagnostic criteria are either objective or subjective. When specific imaging and field criteria are chosen, there is the possibility that expert clinicians would differ on those criteria. When subjective expert judgment is the defining rule, one must be concerned that the result is not reproducible by some other group of experts. Clinician diagnostic biases may therefore be embedded in the characteristics of the two groups in this study and only through application of the method to other databases, collected prospectively, and with a variety of diagnostic criteria, will the ultimate value of the method be demonstrated.

A related issue with the glaucoma subjects used to test the SFI is that a significant portion of them has a visual field mean deviation with a value greater than 0 (Figure 5). This would imply that the clinicians making the diagnosis of glaucoma were likely using optic disc or retinal nerve fiber layer examination to define the presence of glaucoma. This group of glaucoma patients with above average field sensitivity could be explained either by the fact that optic disc change can precede visual field loss[17] or by mis-diagnosis by the examining clinician. The latter option again points out the ambiguity caused by relying on "expert" clinicians to define the presence or absence of a disease and is something the SFI might help overcome.

## Conclusions

Given the widespread availability of quantitative measures of optic nerve structure and function, clinicians now must combine those measures in a subjective manner. We believe there is a compelling need to develop computational models that unify visual field and optic nerve imaging data, based on known anatomy, to improve glaucoma diagnosis. The features of the SFI were intended to explicitly model our understanding of changes in optic nerve structure and function in glaucoma. Furthermore, the approach we have defined is not restricted to the testing modalities used here. The SFI could just as easily be calculated using other tests of visual function (frequency doubling perimetry, short wavelength perimetry, etc.) and other tests of optic nerve structure (optical coherence tomography, scanning laser polarimetry). Use of spectral domain OCT is particularly promising as it may be possible to image the nerve fiber layer in each retinal region corresponding to a visual field location. Relating structure and function in this way would avoid the use of the field-to-disc maps discussed above.

Based on the characteristics of the SFI in eyes with glaucoma and those merely suspected of having disease, we believe further work is warranted and that this approach, or one like it, might provide a new standard for diagnosing glaucoma.

## Competing interests

The authors declare that they have no competing interests.

## Authors' contributions

MB contributed to the design the SFI method and the study, analyzed the data, and drafted and approved the manuscript. HQ contributed to the design of the SFI method and the study, and drafted and approved the manuscript.

## Pre-publication history

The pre-publication history for this paper can be accessed here:

http://www.biomedcentral.com/1471-2415/11/6/prepub
